# Doxorubicin promotes breast cancer cell migration and invasion via DCAF13

**DOI:** 10.1002/2211-5463.13330

**Published:** 2021-12-05

**Authors:** Zhaoran Sun, Dongmei Zhou, Jinkui Yang, Daoyong Zhang

**Affiliations:** ^1^ Jiangsu Key Laboratory of Brain Disease and Bioinformation Research Center for Biochemistry and Molecular Biology Xuzhou Medical University China; ^2^ Graduate School of Capital Medical University Beijing China; ^3^ Department of Endocrinology Affiliated Hospital of Xuzhou Medical University China; ^4^ Department of Endocrinology Beijing Tongren Hospital Capital Medical University Beijing China

**Keywords:** breast cancer, chemotherapy, DCAF13, doxorubicin, EMT, metastasis

## Abstract

DDB1 and CUL4 associated factor 13 (DCAF13) is a substrate receptor in the CUL4‐DDB1 E3 ligase, and its expression is associated with the prognosis of certain cancers. In the present study, we report evidence that DCAF13 is aberrantly overexpressed in human breast cancer and its expression is positively associated with cancer progression. Further analysis showed that the DCAF13 expression level is significantly higher in triple‐negative breast cancer compared to non‐triple‐negative breast cancer, indicating a positive correlation between its expression and the aggressiveness of breast cancer. Subsequent studies revealed that DCAF13 regulates cancer cell migration, invasion and epithelial–mesenchymal transition in human breast cancer, whereas it has no significant impact on breast cancer cell proliferation, cell cycle progressionor apoptosis. Taken together, our results demonstrate that DCAF13 promotes the epithelial–mesenchymal transition in human breast cancer cells, indicating an involvement in breast cancer metastasis. Furthermore, we report that doxorubicin, a widely used chemotherapy drug, increases DCAF13 expression in breast cancer cells, leading to enhanced cancer cell migration and invasion. These results suggest that doxorubicin chemotherapy may increase the risk of metastasis of drug‐resistant breast cancer cells, and future therapeutics targeting DCAF13 may help reduce the risk, especially for patients undergoing chemotherapy.

## Graphical abstract

DCAF13 is a substrate receptor in the CUL4‐DDB1 E3 ligase. We report that doxorubicin, a widely used chemotherapy drug, increases DCAF13 expression in breast cancer cells, leading to enhanced cancer cell migration and invasion, suggesting that doxorubicin may increase the risk of metastasis of drug‐resistant breast cancer cells, and future therapeutics targeting DCAF13 may help reduce the risk.

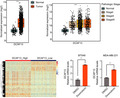

**Abbreviations**
DCAF: DDB1 and CUL4 associated factor
EMT: epithelial–mesenchymal transition
GSEA: gene set enrichment analysis
TNBC: triple‐negative breast cancer
TCGA: The Cancer Genome Atlas
CCK‐8: cell counting kit‐8
RBP: RNA‐binding protein

The DDB1 and CUL4 associated factors (DCAFs) comprise a family of WD40 domain‐containing proteins, serving as the substrate receptors in the CUL4‐DDB1 E3 ligase. DCAFs are connected to CUL4 with DDB1 as the adaptor, assembling into as many as 90 E3 complexes [1]. DCAFs are the main determinant for the substrate specificity of the CUL4‐DDB1 E3 ligase, bridging the proteins targeted for polyubiquitination and the CUL4‐DDB1 E3 ligase. DCAF13 is a specific member of the family of DCAFs.

Although the cellular substrates recognized by the DCAF13 have not been fully uncovered, a series of recent studies indicate that DCAF13 regulates a few biological processes by targeting some key players for polyubiquitination and proteasomal degradation. In human embryos, DCAF13 is expressed from eight‐cell stage to morula stage and the knockout of DCAF13 leads to preimplantation lethality. Interestingly, the H3K9 trimethylation levels are dramatically elevated in DCAF13‐null embryos. H3K9 trimethylation is a key barrier to gene expression reprogramming in early embryo development. Mechanistically, DCAF13 recognizes SUV39H1, a H3K9 methyl transferase, and targets it for polyubiquitination and proteasomal degradation, thus promoting H3K9 trimethylation removal [2]. In oocytes, DCAF13 was identified as a nucleolar protein and an important component of the rRNA‐processing complex. Conditional knockout of DCAF13 results in defects in oocyte development and female fertility. Further analysis revealed that DCAF13 is implicated in 18S rRNA processing in growing oocytes [3].

A few studies also suggest that DCAF13 is an important player in cancers. In osteosarcoma cells, CRL4B^DCAF13^ E3 ligase has been shown to specifically target the tumor suppressor PTEN (phosphatase and tensin homolog deleted on chromosome 10) for polyubiquitination and proteasomal degradation [4]. The *DCAF13* gene was also found to be amplified in 14.7% of cases of hepatocellular carcinoma and its expression was upregulated in hepatocellular carcinoma. DCAF13 expression is positively correlated with advanced hepatocellular carcinoma grade and closely associated with poorer survival [5]. Besides hepatocellular carcinoma, DCAF13 was also shown to be of prognostic value in lung adenocarcinoma [6].

In the present study, we show that DCAF13 is aberrantly overexpressed in human breast cancer. Further analysis shows that DCAF13 promotes epithelial–mesenchymal transition (EMT) in breast cells, whereas it does not have significant impact on breast cell proliferation, cell cycle progression and apoptosis.

## Materials and methods

### Gene expression and clinical phenotype data source

mRNA expression data and clinical phenotype data of breast cancer from The Cancer Genome Atlas (TCGA) were obtained from the TCGA project (http://portal.gdc.cancer.gov), including 1097 primary breast cancer samples and 113 normal breast tissue samples. For the differential gene expression analysis and gene set enrichment analysis (GSEA), breast cancer samples were categorized into DCAF13‐high and DCAF13‐low groups based on the median DCAF13 expression level in primary breast cancer samples. The differential gene expression analysis was performed using deseq2 (https://bioconductor.org/packages/release/bioc/html/DESeq2.html), version 1.26.0, to identify differentially expressed genes between the DCAF13‐high and DCAF13‐low groups. GSEA was performed with the Bioconductor package clusterprofiler (https://bioconductor.org/packages/release/bioc/html/clusterProfiler.html), version 3.14.0, for the Hallmark gene sets from the GSEA MSigDB (https://www.gsea‐msigdb.org/gsea/msigdb/).

### Cell culture and gene silencing

Breast cancer cell line BT549 and MDA‐MB‐231 were originally purchased from the ATCC (Manassas, VA, USA). BT549 cells were cultured in RPMI 1640 medium (Invitrogen, Waltham, MA, USA) supplemented with 10% FBS. MDA‐MB‐231 cells were cultured in L15 medium (Invitrogen) supplemented with 10% FBS. The short hairpin RNAs targeting DCAF13 and control scrambled sequence were: DCAF13‐sh1, CGAATCTTTCCTGTAGACAAA; DCAF13‐sh2, TGGGATTGATCATCACTGGAA; and shControl, TTCTCCGAACGTGTCACGT. Lentivirus preparation has been described previously [7]. To silence DCAF13 expression, the lentivirus expressing DCAF13‐sh1, DCAF13‐sh2 or shControl was used to infect BT549 cells and MDA‐MB‐231 cells followed by selection with puromycin for 2 weeks. The knockdown efficiency was then evaluated by a quantitative real‐time PCR.

### Doxorubicin treatment and quantitative real‐time PCR

To evaluate the effect of doxorubicin treatment on DCAF13 expression in breast cancer cells, MDA‐MB‐231 cells were treated with 100 nm doxorubicin (catalog. no. HY‐15142; MedChemExpress, Monmouth Junction, NJ, USA) for 48 h, and BT549 cells were treated with 25 nm doxorubicin for 24 h, respectively. Total RNA was extracted from cells with TRIzol (Invitrogen) in accordance with the manufacturer’s instructions. cDNA was generated by reverse transcription using HiScript III RT SuperMix for qPCR (+g DNA wiper) (catalog. no. R123‐01; Vazyme, Nanjing, China). Quantitative real‐time PCR was performed on an ABI‐7500 using TB Green™ Premix Ex Taq™ (Tli RNaseH Plus) (catalog. no. RR420A; Takara, Shiga, Japan). Primers used in quantitative real‐time PCR were: β‐actin forward, 5′‐CTGGAACGGTGAAGGTGACA‐3′, β‐actin reverse: 5′‐AAGGGACTTCCTGTAACAACGCA‐3′; DCAF13 forward, 5′‐GGACAGCAAGTAGACATTTGGG‐3′, DCAF13 reverse: 5′‐CAAAGGAGTAGCTTGCCTCA‐3′. β‐actin was used as internal control for normalization.

### Western blotting, immunofluorescence staining and antibodies

For western blotting assays, whole‐cell extracts were denatured, separated on SDS/PAGE gels and transferred to poly(vinylidene difluoride) membranes. After blocking in 5% (w/v) non‐fat dry milk in Tris‐buffered saline with Tween, membranes were probed with primary antibodies overnight at 4 °C. For immunofluorescence staining, cells were fixed in 4% paraformaldehyde in PBS for 15 min at room temperature, permeabilized with 0.5% triton x‐100 in PBS for 5 min, blocked for 1 h at room temperature with 10% normal goat serum in PBS and incubated overnight at 4 °C with primary antibodies. Primary antibodies used were: anti‐β‐catenin (catalog. no. 51067‐2‐AP; ProteinTech, Rosemont, IL, USA), anti‐fibronectin (catalog. no. 15613‐1‐AP; ProteinTech) and anti‐β‐tubulin (catalog. no. 66240‐1‐Ig; ProteinTech).

### Proliferation, wound healing and transwell assays

Cell counting kit‐8 (CCK‐8) proliferation, wound healing and transwell cell invasion assays were performed as described previously [7]. Three independent experiments were performed for the statistical analysis.

### Flow cytometry analysis

Cells were grown in a six‐well plate to 80% confluence. Then, trypsin without EDTA was used to treat cells to obtain single cell suspension. For cell cycle analysis, single cell suspension (1 × 10^6^ cells·mL^−1^) was fixed in 70% ice‐cold ethanol overnight, and then washed and stained in dark in propidium iodide/RNase staining buffer at room temperature for 30 min in accordance with the manufacturer’s instructions (catalog. no. KGA512; KeyGen, Nanjing, China). For apoptosis analysis, cell suspension (5 × 10^5^ cells·mL^−1^) in 1 × binding buffer was stained with annexin V‐kFluor647 and propidium iodide in accordance with the manufacturer’s instructions (catalog. no. KGAV115; KeyGen). Three independent experiments were performed for statistical analysis.

## Results

### DCAF13 expression is aberrantly elevated in breast cancer

**Fig. 1:**
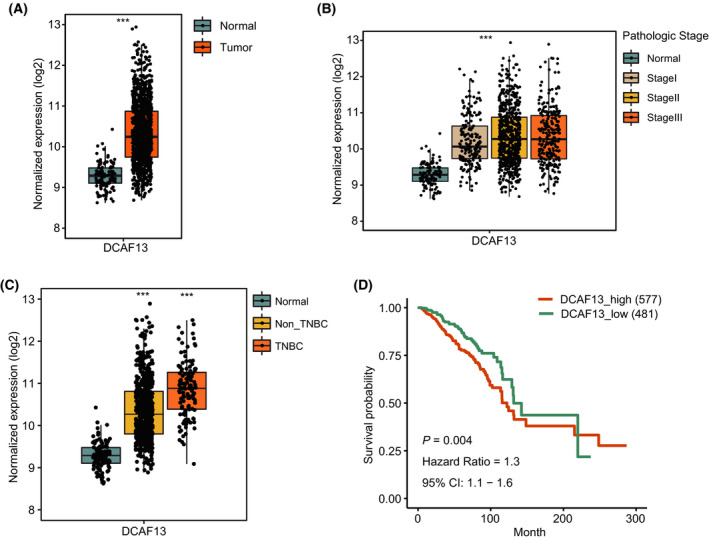
DCAF13 expression is aberrantly elevated in breast cancer. (A) DCAF13 expression was analyzed in human breast cancer samples (*n* = 1097) and normal breast tissue samples (*n* = 113). (B) DCAF13 expression was positively correlated with cancer progression in human breast cancer. Cases lacking pathologic stage information were not included in these analyses. (C) DCAF13 expression level is significantly higher in human TNBC. DCAF13 expression was analyzed in TNBC samples (*n* = 122), non‐TNBC samples (*n* = 617) and normal breast tissue samples (*n* = 113). ***P < 0.001, unpaired *t*‐test. (D) High DCAF13 expression is associated with poor prognosis of breast cancer patients. A log‐rank test was performed to assess the difference between the survival curves of the DCAF13_high group and the DCAF13_low group. The number of cases analyzed in each group is indicated.

To evaluate the DCAF13 expression in human breast cancer, we downloaded RNA‐sequencing data from TCGA (http://portal.gdc.cancer.gov/). In total, 1097 primary breast cancer samples and 113 normal breast tissue samples were included in the analysis. The results showed that DCAF13 expression was aberrantly upregulated in breast cancer samples compared to normal breast tissue samples (Fig. 1A). Furthermore, DCAF13 expression was positively correlated with advanced pathologic stages, indicating its correlation with cancer progression (Fig. 1B).

Triple‐negative breast cancer (TNBC) is an aggressive subtype of breast cancer with higher mortality rate. We also split the breast cancer samples into TNBC and non‐TNBC groups and analyzed DCAF13 expression separately in both groups, as well as in normal breast tissue samples. As shown in Fig. 1C, DCAF13 expression level was higher in both non‐TNBC and TNBC samples compared to normal breast tissue samples. Interestingly, DCAF13 expression level was significantly higher in TNBC samples compared to non‐TNBC samples, suggesting a positive correlation between DCAF13 expression and breast cancer aggressiveness.

To evaluate the prognostic value of DCAF13 expression in human breast cancer, we performed a log‐rank test to assess the difference between the survival curves of the DCAF13_high group and the DCAF13_low group. The optimal cut‐off value was determined by the surv_cutoff function of the r package survminer (https://cran.r‐project.org/package=survminer). The result showed that high DCAF13 expression is associated with a poor overall survival probability in patients with breast cancer (Fig. 1D).

### DCAF13 promotes EMT in human breast cancer

**Fig. 2:**
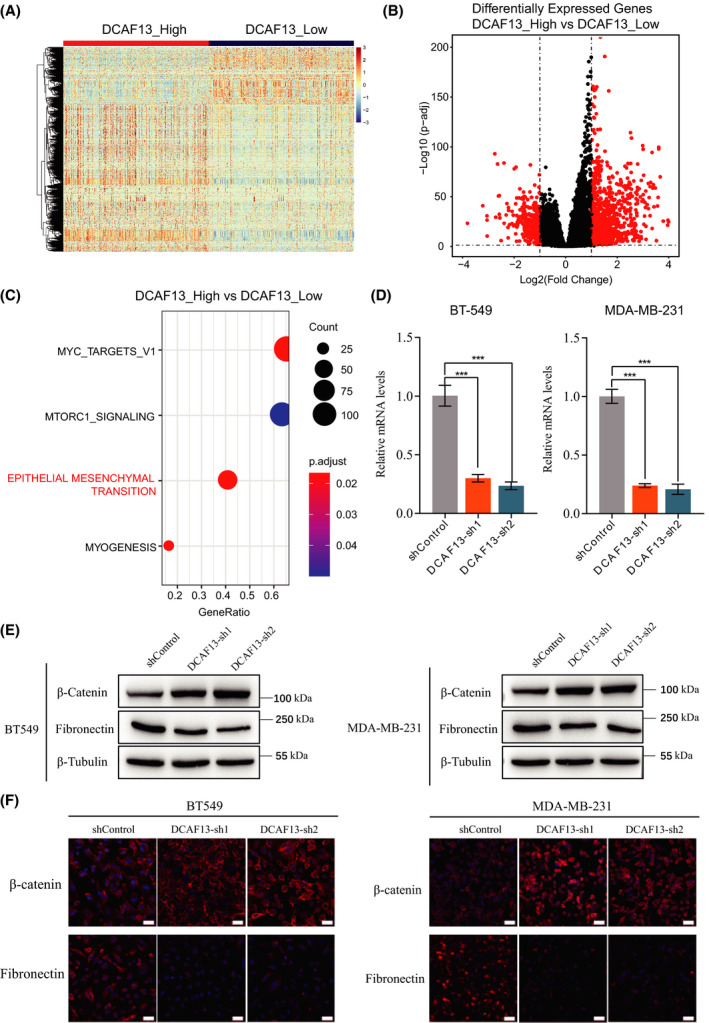
DCAF13 promotes EMT in human breast cancer. (A) Hierarchical cluster analysis of differentially expressed genes (|logFC| > 1 and *P*_adj_ < 0.001) between DCAF13_high and DCAF13_low breast cancer samples. (B) The volcano plot of differential gene expression between DCAF13_high and DCAF13_low breast cancer samples. The horizontal dashed line marks the threshold (*P*_adj_ < 0.05) for defining a gene as significantly upregulated or downregulated. The vertical dashed lines represent two‐fold differences in expression. Significantly differentially expressed genes are shown as red dots (*P*_adj_ < 0.05 and |log2FC| > 1). (C) GSEA analysis was performed to identify the pathways altered in DCAF13_high samples compared to DCAF13_low samples. (D) A quantitative PCR was carried out to verify DCAF13 knockdown efficiency in BT549 cells and MDA‐MB‐231 cells. Data are presented as the mean ± SD. (*n* = 3 independent experiments). ***P < 0.001, unpaired *t*‐test. (E,F) Western blot and immunofluorescence staining assays were carried out to examine the effect of DCAF13 knockdown on EMT marker gene expression. Scale bar = 50 μm.

Next, we attempted to explore the cellular processes that are regulated by DCAF13 in human breast cancer. To this end, we split the breast cancer samples into DCAF13‐high and DCAF13‐low groups based on the median DCAF13 expression level. Differential gene expression analysis was performed using deseq2, version 1.26.0, to identify differentially expressed genes between the DCAF13‐high and DCAF13‐low groups. We then carried out GSEA with the Bioconductor package clusterProfiler, version 3.14.0, for the Hallmark gene sets from GSEA MSigDB. As shown in Fig. 2A,B, we observed distinct gene expression patterns in the two groups. The GSEA results showed that four gene sets were significantly enriched, including Epithelial‐Mesenchymal Transition, Myc‐Targets, MTORC1_signaling and Myogenesis (Fig. 2C).

EMT is a critical mechanism for the acquisition of invasiveness by epithelial cancer cells and closely associated cancer metastasis [8]. The finding of EMT gene set enrichment prompted us to further explore the regulatory role of DCAF13 in the EMT of breast cancer cells. To this end, we first knocked down DCAF13 expression in breast cancer cell lines, BT549 and MDA‐MB‐231, and then examined the effect of DCAF13 knockdown on EMT gene expression. As shown in Fig. 2D–F, the epithelial cell marker β‐catenin was upregulated, whereas the mesenchymal cell marker fibronectin was downregulated upon DCAF13 knockdown, suggesting that DCAF13 promotes EMT in human breast cancer.

### DCAF13 knockdown inhibits migration and invasion of breast cancer cells

**Fig. 3:**
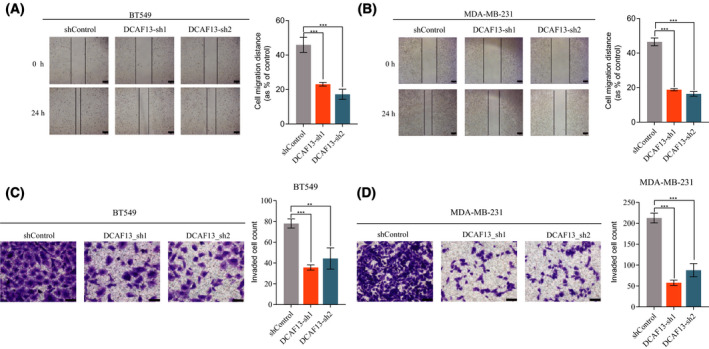
DCAF13 depletion inhibits breast cancer cell migration and invasion. (A,B) DCAF13 depletion inhibits breast cancer cell migration. Wound healing assays were carried out to assess the effect of DCAF13 depletion on breast cancer cell migration. Three independent experiments were carried out and the results were quantified and represented as the mean ± SD. ***P < 0.001, unpaired *t*‐test. Scale bar = 100 μm. (C,D) DCAF13 depletion inhibits breast cancer cell invasion. Transwell cell invasion assays were performed to evaluate the effect of DCAF13 depletion on breast cancer cell invasion. Three independent experiments were carried out and the results were quantified and represented as the mean ± SD. **P < 0.01, ***P < 0.001, unpaired *t*‐test. Scale bar = 100 μm.

**Fig. 4:**
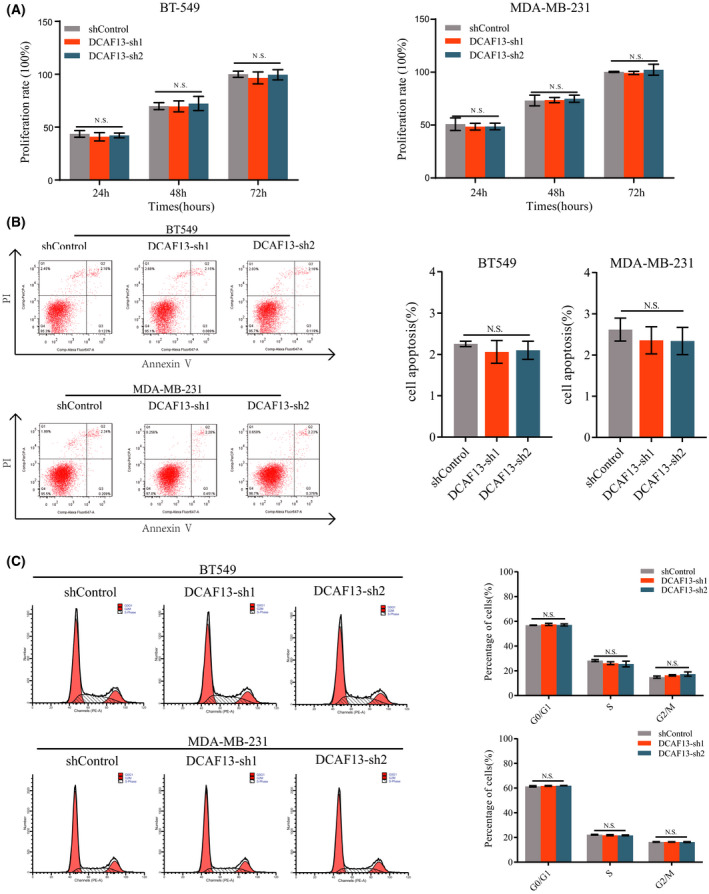
DCAF13 depletion has no significant impact on breast cancer cell proliferation, cell cycle progression and apoptosis. (A) A CCK‐8 assay was employed to evaluate the effect of DCAF13 depletion on breast cancer cell proliferation. (B,C) Flow cytometry analyses were carried out to evaluate the effect of DCAF13 depletion on cell cycle progression and apoptosis of breast cancer cells. For statistical analyses, three independent experiments were carried out and the results were quantified and represented as the mean ± SD. An unpaired *t*‐test was performed to examine the statistical significance.

Given the fact that EMT is closely associated with cancer cell invasiveness and cancer metastasis, we next investigated the impact of DCAF13 knockdown on breast cancer cell migration and invasion. A wound healing assay was carried out to assess the change in breast cancer cell migration ability upon DCAF13 knockdown. As shown in Fig. 3A,B, DCAF13 knockdown dramatically reduced the migration ability of breast cancer cells. We also carried out a transwell cell invasion assay to examine the effect of DCAF13 knockdown on breast cancer cell invasion ability. As shown in Fig. 3C,D, we also observed a dramatic decrease in the invasion ability of breast cancer cells. Together, these results demonstrate that DCAF13 regulates EMT gene expression in breast cancer and thus regulates breast cancer metastasis. We also investigated the regulatory role of DCAF13 in cell proliferation, cell cycle progression and apoptosis of breast cancer cells. As shown in Fig. 4, we did not observe any significant impact of DCAF13 silencing on breast cell proliferation, cell cycle progression and apoptosis, suggesting that DCAF13 may not regulate primary tumor growth of breast cancer.

### Doxorubicin treatment promotes breast cancer cell migration and invasion via DCAF13

**Fig. 5:**
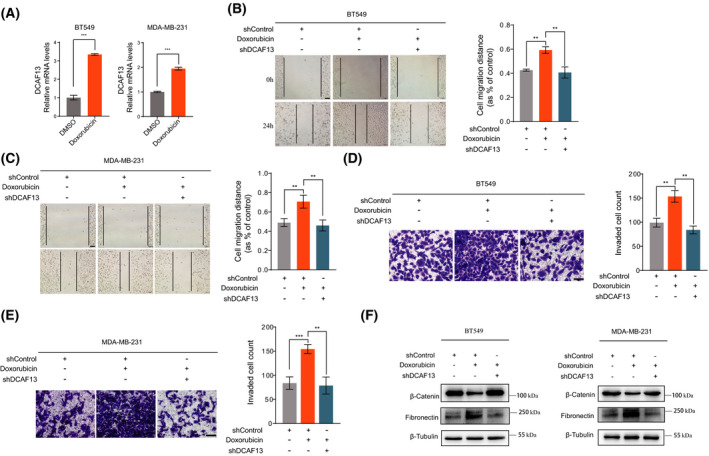
Doxorubicin promotes breast cancer cell migration, invasion and EMT via DCAF13. (A) Doxorubicin treatment increased DCAF13 expression in breast cancer cells. BT549 cells and MDA‐MB‐231 cells were treated with doxorubicin and total RNA was extracted for real‐time PCR analysis. Three independent experiments were carried out and the results were quantified and represented as the mean ± SD. ***P < 0.001, unpaired *t*‐test. (B,C) Doxorubicin promotes breast cancer cell migration via DCAF13. Wound healing assays were carried out to assess the effect of doxorubicin treatment on breast cancer cell migration and the involvement of DCAF13. Three independent experiments were carried out and the results were quantified and represented as the mean ± SD. **P < 0.01, unpaired *t*‐test. Scale bar = 100 μm. (D,E) Doxorubicin promotes breast cancer cell invasion via DCAF13. Transwell cell invasion assays were performed to assess the effect of doxorubicin treatment on breast cancer cell invasion and the involvement of DCAF13. Three independent experiments were carried out and the results were quantified and represented as the mean ± SD. **P < 0.01, ***P < 0.001, unpaired *t*‐test. Scale bar = 100 μm. (F) Doxorubicin promotes the EMT in breast cancer cells via DCAF13. A western blot assay was performed to examine the effect of doxorubicin treatment on EMT marker gene expression and the involvement of DCAF13.

Given the promoting role of DCAF13 in breast cancer metastasis, we were interested in investigating whether chemotherapy drug treatment would alter the expression of DCAF13 in breast cancer cells, which may lead to enhanced or impeded breast cancer metastasis depending on how DCAF13 expression is altered. To this end, we treated BT549 cells and MDA‐MB‐231 cells with doxorubicin and extracted total RNA for a quantitative real‐time PCR. Doxorubicin is a commonly used chemotherapy drug for breast cancer patients. The result showed that doxorubicin treatment dramatically increased DCAF13 expression in breast cancer cells (Fig. 5A).

To further investigate whether the upregulation of DCAF13 by doxorubicin treatment may lead to enhanced breast cancer metastasis, we performed wound healing and transwell cell invasion assays. As shown in Fig. 5B–E, doxorubicin treatment significantly increased the migration and invasion ability of breast cancer cells. This effect is mediated by DCAF13 because DCAF13 silencing abrogated this effect (Fig. 5B–E). Furthermore, we also evaluated the effect of doxorubicin treatment on the expression of the EMT markers. As shown in Fig. 5F, β‐catenin was downregulated and fibronectin was upregulated upon doxorubicin treatment, indicating that doxorubicin treatment promotes EMT of breast cancer cells. Again, this effect was also mediated by DCAF13 because DCAF13 silencing suppressed doxorubicin‐induced EMT of breast cancer cells (Fig. 5F).

These results are of particular importance with respect to the clinical treatment of breast cancer patients because chemotherapy appears to be a double‐edged sword for treating breast cancer patients. On the one hand, drug treatment of breast cancer patients kills primary cancer cells. On the other hand, it increases the probability of metastasis of drug‐resistant cancer cells by increasing DCAF13 expression. Future therapeutics targeting DCAF13 may help reduce the risk of breast cancer metastasis, especially for patients undergoing chemotherapy.

## Discussion

DCAF13 is a substrate receptor in the CUL4‐DDB1 E3 ligase. DCAF13 has been shown to be involved in some biological processes and a few types of human cancer. In the present study, we report evidence showing that DCAF13 is aberrantly overexpressed in human breast cancer and its expression positively associates with cancer progression. Further analysis showed that DCAF13 expression level is significantly higher in TNBC compared to non‐TNBC, indicating its positive correlation with breast cancer aggressiveness. By analyzing the RNA‐sequencing data, we found that DCAF13 might regulate EMT in human breast cancer. The results of western blotting and immunofluorescence staining assays demonstrate that DCAF13 promotes EMT in human breast cancer. This conclusion is further supported by the results of subsequent cell migration and cell invasion assays. However, DCAF13 does not regulate breast cancer cell proliferation, cell cycle progression and apoptosis. Taken together, our results demonstrate a promoting role of DCAF13 in the EMT process of human breast cancer cells. In line with our findings, a recent study reports that DCAF13 promotes triple‐negative breast cancer metastasis [9]. Mechanistically, DCAF13 acts as a RNA‐binding protein (RBP) in TNBC to bind to DTX3 mRNA and target it for degradation [9].

Chemotherapy is being widely used in treating many types of cancer and is one of the most effective ways, along with surgical resection and radiotherapy. Although chemotherapy considerably improves survival in cancer patients, drug resistance has limited success. Over the course of chemotherapy, cancer cells gradually acquire resistance to the drugs used in the chemotherapy, which eventually leads to relapse [10]. Doxorubicin is an efficacious chemotherapy drug used in the treatment of various types of cancer, including breast cancer, lung cancer, glioblastoma, glioma and thyroid cancers [11, 12, 13, 14]. Although doxorubicin is a potent therapeutic agent, it has adverse side effects, such as cardiotoxicity [15, 16]. In present study, we report evidence demonstrating that doxorubicin treatment promotes breast cancer cell migration and invasion by upregulating DCAF13. This is of particular importance because we identified a novel factor that mediates an undesirable outcome of doxorubicin chemotherapy of breast cancer patients. DCAF13 may serve as a therapeutic target for reducing the risk of breast cancer metastasis, especially for patients undergoing doxorubicin chemotherapy.

As a substrate receptor in the CUL4‐DDB1 E3 ligase, the cellular functions of DCAF13 are determined by the substrates that DCAF13 recognizes and the targets for polyubiquitination and proteasomal degradation. However, very few DCAF13 substrates have been identified so far [2]. Although, in the present study, we demonstrate that DCAF13 promotes EMT in breast cancer and thus promotes metastasis, we do not yet know what substrates of DCAF13 are involved in this process. Further screenings for DCAF13 substrates in breast cancer are needed in this regard. Alternatively, DCAF13 may function as a RBP targeting the mRNAs of crucial EMT repressors for degradation to promote EMT in breast cancer.

## Conclusions

In the present study, we report evidence revealing that DCAF13 is aberrantly overexpressed in human breast cancer and its expression positively associates with cancer progression. Further analysis demonstrates that DCAF13 promotes EMT in human breast cancer cells, whereas it has no regulatory roles in breast cell proliferation, cell cycle progression and apoptosis. Furthermore, we demonstrate that doxorubicin treatment increases DCAF13 expression in breast cancer cells, thereby promoting cancer cell migration and invasion, indicating that doxorubicin chemotherapy may increase the risk of metastasis of drug‐resistant breast cancer cells by increasing DCAF13 expression. Future therapeutics targeting DCAF13 may help reduce the risk of breast cancer metastasis, especially for patients undergoing chemotherapy.

## Conflict of interests

The authors declare no conflict of interest.

## Author contributions

JY and DZhang designed and supervised the study. ZS and DZhou performed the research. ZS, DZhou and DZhang analyzed the data. DZhang wrote the paper. All authors have read and approved the final version of the manuscript submitted for publication.

## Data Availability

The data that support the findings of this study are available from the corresponding author upon reasonable request.
